# Cancer Development, Progression, and Therapy: An Epigenetic Overview

**DOI:** 10.3390/ijms141021087

**Published:** 2013-10-21

**Authors:** Sibaji Sarkar, Garrick Horn, Kimberly Moulton, Anuja Oza, Shannon Byler, Shannon Kokolus, McKenna Longacre

**Affiliations:** Cancer Center, L913, Department of Medicine, Boston University School of Medicine, 72 East Concord Street, Boston, MA 02118, USA; E-Mails: garrick@bu.edu (G.H.); moulton@bu.edu (K.M.); anujaoza@bu.edu (A.O.); sbyler@bu.edu (S.B.); kokolus@gmail.com (S.K.); mckennalongacre@gmail.com (M.L.)

**Keywords:** cancer, epigenetics, methylation, demethylation, hydroxymethylation, apoptosis, microRNA, metastasis, epithelial-mesenchymal transition (EMT), therapeutics

## Abstract

Carcinogenesis involves uncontrolled cell growth, which follows the activation of oncogenes and/or the deactivation of tumor suppression genes. Metastasis requires down-regulation of cell adhesion receptors necessary for tissue-specific, cell–cell attachment, as well as up-regulation of receptors that enhance cell motility. Epigenetic changes, including histone modifications, DNA methylation, and DNA hydroxymethylation, can modify these characteristics. Targets for these epigenetic changes include signaling pathways that regulate apoptosis and autophagy, as well as microRNA. We propose that predisposed normal cells convert to cancer progenitor cells that, after growing, undergo an epithelial-mesenchymal transition. This process, which is partially under epigenetic control, can create a metastatic form of both progenitor and full-fledged cancer cells, after which metastasis to a distant location may occur. Identification of epigenetic regulatory mechanisms has provided potential therapeutic avenues. In particular, epigenetic drugs appear to potentiate the action of traditional therapeutics, often by demethylating and re-expressing tumor suppressor genes to inhibit tumorigenesis. Epigenetic drugs may inhibit both the formation and growth of cancer progenitor cells, thus reducing the recurrence of cancer. Adopting epigenetic alteration as a new hallmark of cancer is a logical and necessary step that will further encourage the development of novel epigenetic biomarkers and therapeutics.

## 1. Introduction

According to the original hallmarks of cancer, six different capabilities lead to the development and progression of cancers [1]. Developments in the conceptual understanding of cancer biology in the past decade have led to the recent suggestion that reprogramming of metabolism and evasion of immune destruction be accepted as additional hallmarks [2]. We propose that epigenetic alteration should be considered another hallmark of cancer, and thus an additional focus for study for the next generation of cancer therapies.

Cancer is characterized by uncontrolled cell growth and acquisition of metastatic properties. In most cases, activation of oncogenes and/or deactivation of tumor suppressor genes lead to uncontrolled cell cycle progression and inactivation of apoptotic mechanisms. As opposed to benign tumors, malignant cancers acquire metastasis, which occurs in part due to the down-regulation of cell adhesion receptors necessary for tissue-specific cell–cell attachment, and up-regulation of receptors that enhance cell motility. In addition, activation of membrane metalloproteases provides a physical pathway for metastatic cancer cells to spread. There are different mechanisms by which these genetic and cellular changes occur. The canonical mechanisms are mutation, chromosomal translocation or deletion, and dysregulated expression or activity of signaling pathways. These events may activate genes that promote dysregulated cell cycling and/or inactivate apoptotic pathways. These processes are well described in the existing literature, and numerous excellent reviews are available on each topic [3,4].

**Figure 1:**
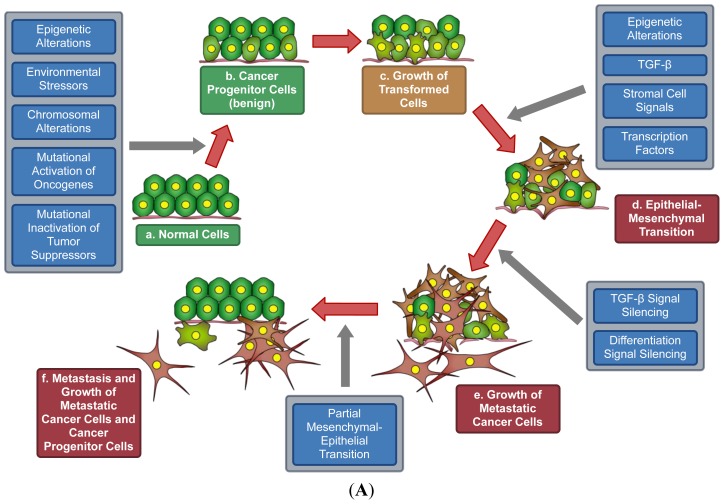

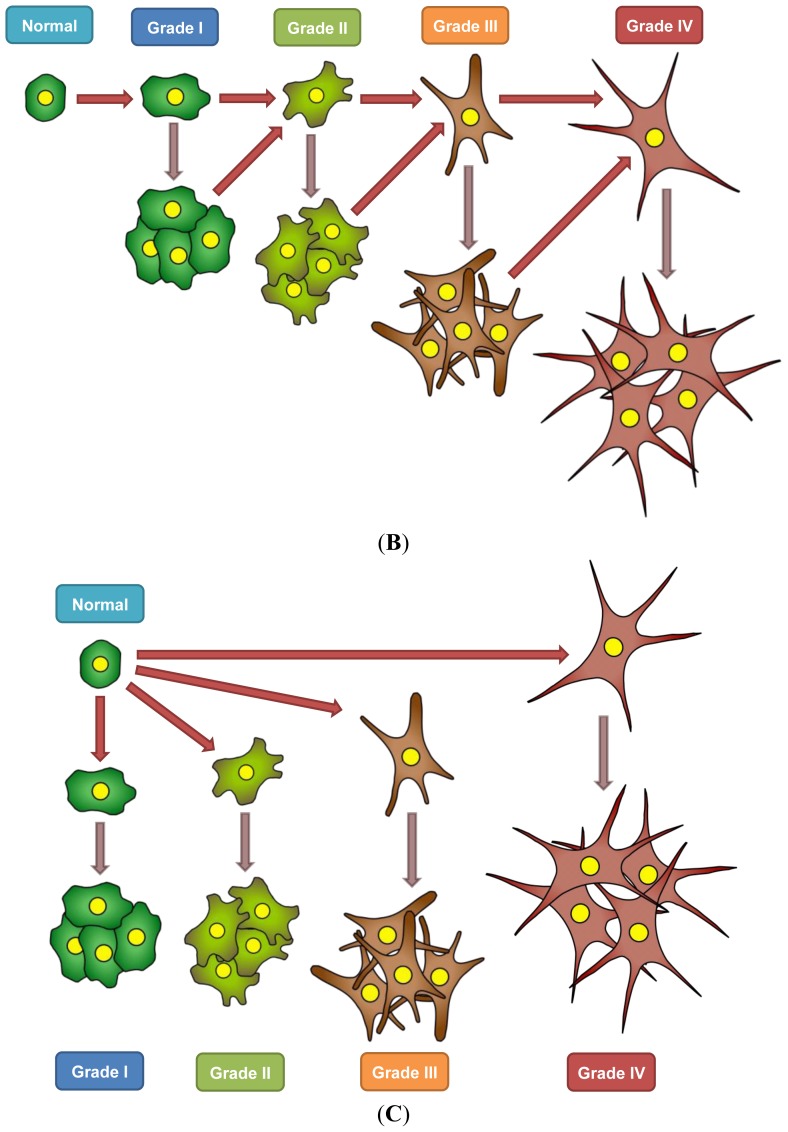
(**A**) Cancer progenitor cells and progression of metastatic cancer. **a**: hexagons with yellow dots represent normal cells; **b**: faded green, distorted hexagons with yellow dots represent cancer progenitor cells; **c**: progenitor cells are increasing in number; **d**: star-like brown cells represent the metastatic form of cancer cells, a mixed population of progenitor and adult cells; **e**: overgrowth of metastatic cells; **f**: both metastatic and adult progenitor cells leave site. Progression: Cancer progenitor cells develop from normal cells (**a** to **b**); After growth (**b** to **c**), they undergo EMT (**c** to **d**); Differentiation signals decrease and growth signals increase, producing a combination of progenitor and adult metastatic cancer cells (**d** to **e**); After the outgrowth of metastatic cells, translocation to a distant location occurs (**e** to **f**); (**B**) Model for the development of grade-specific cancers. Cancer progenitor cells pause at each grade of differentiation and proliferate from that grade while maintaining the ability to differentiate further; and (**C**) Model of the development of grade-specific cancers. Some cells progress further through differentiation than others, stop differentiation, and then proliferate, giving rise to clonal populations of cancer cells at distinct grades.

The role of epigenetics in carcinogenesis is less well defined. Recent studies suggest that epigenetic alteration may be another hallmark of cancer due to its role in the generation of cancer progenitor cells and subsequent initiation of carcinogenesis. Such modifications are covalent, and may affect histones or DNA residues. We recently suggested a new paradigm for cancer progression in which epigenetic changes play a key role in the development of these clinically significant cell features [5]. Epigenetic changes can induce pro-cancer characteristics in even mutation-free cells [6]. In this review, we will emphasize the role of epigenetics in carcinogenesis and the potential therapeutics derived from this perspective. We also hypothesize a model for the development of metastatic cancer progenitor cells from non-metastatic progenitor cells (Figure 1).

## 2. DNA Methylation

Epigenetic changes are alterations in gene expression, independent of changes in DNA sequence. Many epigenetic modifications, such as DNA methylation and hydroxymethylation, histone acetylation and methylation, and changes in small noncoding RNAs, have profound effects on gene expression. DNA methylation at CpG islands has been shown to silence gene expression by interfering with transcriptional machinery [7,8].

For decades, cancer development was attributed to purely genetic mechanisms, but a growing body of evidence has revealed that much of its complexity can be directly attributed to epigenetics [9]. Cell cycle progression and differentiation are tightly controlled processes with complex regulatory mechanisms, and epigenetic changes can have profound effects on these processes. Cell cycle regulators, such as p16, p21, p27, and p53, are silenced by methylation in many cancers [10–12]. RAR-β2, one of the important initiators of differentiation, is also silenced in many forms of cancer [9,13–15]. Furthermore, the maternally imprinted pro-apoptotic gene, *ARHI*, was recently discovered to be silenced by methylation in breast and ovarian cancer cells. Its paternal expression is downregulated via methylation silencing in ovarian and breast cancer, causing loss of heterozygosity (LOH) [16]. The demethylation of silenced tumor suppressor genes may lead to re-expression, leading to cell-cycle inhibition and apoptosis [17].

Another interesting example of epigenetic silencing involves the DNA repair protein *O*^6^*-*methylguanine DNA methyltransferase (MGMT). Hypermethylation of the MGMT promoter is a common event in the initiation of carcinogenesis, as it increases the susceptibility of a cell to DNA damage by alkylating agents [18]. However, this increased susceptibility can be exploited, and there now exists a definitive body of evidence that indicates that tumors exhibiting MGMT promoter hypermethylation are significantly more responsive to alkylating chemotherapeutics, such as temozolomide [19]. A recent meta-analysis of glioblastoma studies concluded that patients with a hypermethylated MGMT promoter status had significantly greater overall survival and progression-free survival than those without a methylated MGMT promoter [20].

**Figure 2:**
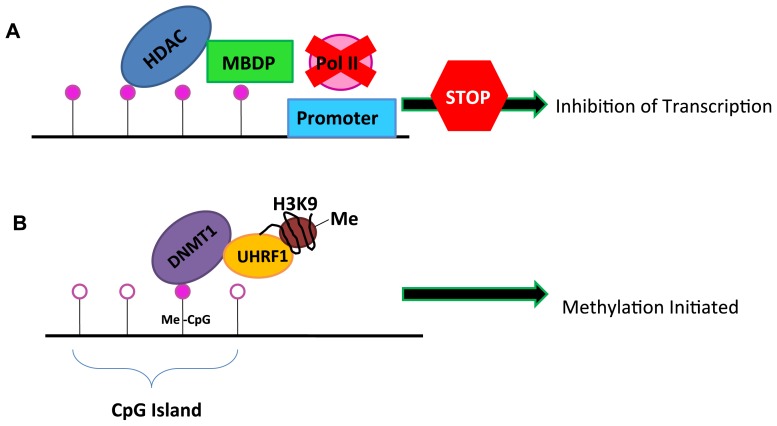
(**A**) Model of inhibition of transcription by methylation of CpG islands in gene promoter regions. HDAC: histone deacetylases; MBDP: methyl binding domain protein; Pol II: RNA polymerase II; and (**B**) Model linking histone methylation with DNA CpG methylation. DNMT1: DNA methyltransferase I; Me-CpG: methylated CpG residue; UHRF1: ubiquitin-like protein containing PHD and RING domains 1; H3K9: histone 3 lysine 9; Me: methylated. Open circles indicate unmethylated CpG residues; closed circles are methylated.

DNA methylation is mediated by DNA-methyltransferases (DNMT). DNMT3a and DNMT3b are responsible for de novo methylation during embryogenesis. DNMT1 has been characterized as the methyltransferase that maintains DNA methylation between cell divisions. DNMT1 is highly expressed in cancer cells [21]. Silencing via DNA methylation at promoter-associated CpG islands involves association of methyl-binding domain proteins (MBDP) and histone deacetylases (HDAC). Binding of these proteins near the promoter region inhibits RNA polymerase 2 binding and thus transcription (Figure 2A). HDAC binding favors a shift to a locally closed chromatin conformation near the regulatory regions of genes. Perhaps it is not surprising then, that in many tumors, HDACs 1, 2, and 6 are overexpressed [22]. Normally, histone-3-K4 (H3K4) methylation is associated with activation of gene expression, and H3K9/H3K27 methylation is associated with inactivation of expression. Aberrant histone modification also plays a role in gene silencing during the development of cancer. Both overexpression and inactivating mutations of H3K4me3/2 histone demethylase family members is hypothesized to contribute to cancer development [23].

A recent study provided evidence that links inhibitory histone modifications (such as H3K9me) to DNA methylation silencing machinery. The protein UHRF1, a ubiquitin-ligase, has been shown to bind a methylated histone residue and subsequently stabilize DNMT1 (Figure 2B). This provides an example of acetylation and methylation processes working in concert to regulate gene expression levels [24].

## 3. Hydroxymethylation

The recent discovery of 5-hydroxymethylcytosine (5hmC) in human tissue has led to significant interest in the potential functions of this novel DNA modification [25]. Computational searches have revealed the mechanism by which 5hmC is generated: TET-mediated hydroxylation of 5-methylcytosine (5mC) to 5hmC [26]. The TET family consists of TET1, TET2, and TET3; all of which contain an alpha-ketoglutarate- and Fe(II)-dependent dioxygenase. Only TET1 and TET3 possess an intrinsic CXXC DNA binding domain. The TET2 CXXC domain appears to have been separated from TET2 by chromosomal rearrangement and is expressed separately as IDAX, which binds to unmethylated CpG-rich regions and negatively regulates TET2 [27].

The roles of the TET family enzymes differ significantly. During development, TET1 is responsible for accumulation of 5hmC at imprinting control regions, while TET2 hydroxylates primarily at pluripotency-related genes [28]. TET3 is most highly expressed in the early post-fertilization period before fusion of the parental pronuclei, and mediates an increase in 5hmC content of the paternal genome, which persists into later cleavage-stage embryos [29,30]. The maternal genome is protected from this process by the pluripotency-associated factor, PGC7, in a yet-uncharacterized fashion [31].

5-Hydroxymethylcytosine levels have been found to be markedly reduced in carcinomas of the prostate, breast, and colon. However, 5mC levels were only modestly decreased, indicating that global DNA hypomethylation could not account for the reduction in 5hmC. Even low histological grade lesions demonstrated a reduction in 5hmC, possibly revealing loss of 5hmC as an early event in carcinogenesis [32].

TET abnormalities and depletion of 5hmC have been reported in many hematopoietic and solid malignancies. TET2 null mutations are found in 22% of acute myeloid leukemia (AML) [33,34]. A TET1 fusion with histone methyltransferase mixed-lineage leukemia (MLL) has also been identified in several cases of AML [35,36]. Decreased 5hmC levels secondary to TET1 expression levels have been identified in liver adenoma, breast carcinoma, lung carcinoma, and pancreas carcinoma [37], and have been clinically correlated with hepatocellular carcinoma tumor size and decreased survival [38]. Decreased levels of 5hmC in non-tumor tissue were also associated with tumor recurrence within one year of surgical resection. Recently, it was reported that reintroduction and overexpression of TET2 in human melanoma cells restores 5hmC content and suppresses tumor invasion and growth [39]. Together, these results imply a tumor suppressor role for TET1 and TET2.

Further support for this hypothesis comes from the discovery of certain gain of function mutations in the isocitrate dehydrogenase (*IDH1* and *IDH2*) pathway, which lead to production of 2-hydroxyglutarate (2-HG) instead of α-ketoglutarate. 2-HG acts as a competitive inhibitor of α-ketoglutarate and perturbs TET enzymatic activity [40,41]. These mutations elicit a cancer phenotype similar to that of TET abnormalities. *IDH1/IDH2* downregulation or mutation has been reported in chondrosarcoma [42], enchondroma [42,43], glioma [44], melanoma [39], and thyroid carcinoma [45,46].

Research has primarily focused on 5hmC as a candidate for a pathway to active DNA demethylation. TET proteins may further oxidize 5hmC to 5-formylcytosine (5fmC) and 5-carboxylcytosine (5cmC), leading to speculation that decarboxylation to 5mC may occur [47]. Indeed, a very recent crystallographic and biochemical study has reported 5cmC decarboxylase activity in fungal isoorotate decarboxylase [48]. This finding will guide searches for analogous or even homologous activity in humans. 5hmC, or 5fmC/5cmC, may also be a signal for the base excision repair-mediated replacement of modified cytosines [49–53].

However, 5hmC has also been demonstrated as a stable DNA modification that persists across several cell divisions, discounting the theory that 5hmC is always efficiently removed [31]. Further experiments have identified 5mC-binding complexes that are disrupted by 5hmC, as well as complexes that specifically bind 5hmC [54,55]. Moreover, 5hmC content is enriched at promoters and gene bodies, as one would expect for a modification with a role in transcriptional regulation [55]. These results support the hypothesis that 5hmC may function not only to release 5mC-binding repressive machinery, but also to recruit machinery with distinct downstream effectors.

## 4. Apoptosis and Autophagy

Epigenetic silencing of tumor suppressor genes promotes tumor progression via inhibition of apoptosis in cancer cells. Apoptosis is a highly regulated process of cell death in the development and maintenance of a normal cell population in mature organisms. Deregulation of apoptosis pathways is thus a key feature of carcinogenesis. There are essentially two pathways of apoptosis: intrinsic and extrinsic. The intrinsic pathway involves a competitive balance between anti-apoptotic Bcl-2 and pro-apoptotic BAX; an excess of BAX permeabilizes the mitochondrial membrane to cytochrome c via Apaf-1 signaling [56]. Cytochrome c activates caspase 3 via caspase 9, triggering mass proteolysis and cell death. This pathway is inhibited by regulators such as XIAP and Bcl-2 family proteins, which are upregulated in many types of cancer [57]. The extrinsic pathway is initiated by cell-surface death receptors, the ligands for which are usually in the TNF-α family. The death receptors activate caspase 8, which further activates caspase 3 via Jun-Kinase (JNK) to cause apoptosis. This pathway is negatively regulated by the proteins FLIP_L_ and FLIP_S_[58].

Hypermethylation and decreased expression of tumor necrosis factor (TNF)-related apoptosis inducing ligand (TRAIL) was seen in many ovarian cancers [59]. TRAIL resistant cells survive longer in cell culture than do cells that express TRAIL. Treating TRAIL resistant cells with AzadC, demethylates this ligand and allows for TRAIL-dependent apoptosis [59]. Downregulation of death receptors is also involved in carcinogenesis. In certain ovarian cancer cell lines, death receptors DR-4 and DR-5 are silenced by methylation [59]. The extrinsic pathway is extensively studied in hematologic cancers, but recent reports suggest that the extrinsic pathway also operates in solid tumors [60]. It is important to appreciate that the intrinsic and extrinsic pathways, although distinct, have significant overlap. Both pathways could be partially regulated by signaling molecules such as Akt, NF-κB, Erk, and p53, which indicates that upstream signaling regulates apoptosis [61].

Dietary chemicals can produce an anti-oxidative effect against carcinogens through epigenetic modifications that affect various pathways [62]. These chemicals directly interact with free radicals and also activate stress pathways, leading to the production of anti-oxidative stress proteins. For example, Genisteine isoflavonoid isolated from soybeans, is a demethylating agent which helps in the re-expression of tumor suppressor genes in certain cancer cells. It is also under investigation as an anti-cancer therapy, though its effects appear to be mild [63]. One of the most important pathways against oxidative damage is Nrf2 signaling, by a mechanism called chemoprevention [62]. Nrf2 knockout mice are more susceptible to chemical carcinogens and inflammation. Other MAP kinase pathways have been identified. Each of these parallel pathways includes JNK and p38 and leads to apoptosis. While the ERK pathway regulates cell growth and differentiation, JNK and p38 are activated when stress, such as UV light, inflammatory cytokines, protein synthesis inhibitors, or DNA damaging agents, is put on the cell [62]. These pathways work to ultimately enhance Nrf2 signaling. Chemotherapeutics make use of similar apoptotic mechanisms to target cancer cells for death [64].

Epigenetic control of autophagy also plays an important role in cell death [61,65]. In cancer cells, epigenetic modifications associated with oncogenes negatively regulate the autophagy, indicating that autophagy is tumor suppressive. These genes include Akt-1, Bcl-2, and Ras [61].

In conclusion, inhibition of natural cell death mechanisms such as apoptosis and autophagy plays an important role in tumor progression by permitting abnormal cell growth. However, the dynamic nature of epigenetic modification leaves the door open to the reversal of cancer-related epigenetic changes by drugs that permit re-expression of pro-apoptotic and pro-autophagy tumor suppressors or cell-cycle regulators.

## 5. MicroRNA

MicroRNAs (miRNAs) are non-coding forms of RNA comprised of around 20 nucleic acids, which function to regulate messenger RNA (mRNA) by binding to the 3′ untranslated region (3′ UTR) of the mRNA and triggering degradation or inhibiting translation. In both mechanisms, an antagonistic relationship exists between miRNA and expression of the target mRNA. Despite specificity in the binding to the 3′ UTR, a given miRNA family may target many different mRNAs [66]. Determining the downstream targets of miRNAs is an active area of research.

MicroRNAs have been implicated in the growth and metastasis of many cancers. Numerous studies over the past decade have detailed the association between expression levels of miRNA and carcinogenesis. A recent study that examined tissue samples from 37 prostate cancer patients found 20 miRNAs that were consistently and similarly dysregulated in tumor tissue when compared to normal tissue. Interestingly, the same study identified distinct miRNA profiles in high- *vs.* low-grade tumors [67]. Another recent study examined only miR-100 expression in prostate cancer and found this particular miRNA to be underexpressed in metastatic *vs.* localized disease [68]. Insight into expression levels of miRNAs in various tumor types and at various disease stages has exploded in recent years, and this copious data has been provided and reviewed elsewhere [69–78].

While much is known about the association between miRNA expression levels and specific cancers, less is understood about the mechanisms governing those associations. Recent studies have attempted to identify miRNA targets and explain how miRNA leads to cancer formation and progression.

MicroRNAs are key regulators of cell cycle proteins. A knockdown study of glioblastoma cells exhibiting high miR-21 levels demonstrated that miR-21 controls p53-mediated apoptosis and cell growth and leads to cell cycle arrest [79]. The transforming growth factor-β (TGF-β) and mitochondrial apoptosis pathways also appear to be inhibited by miR-21. This miRNA is aberrantly expressed in many other cancers, such as high-grade urothelial carcinoma of the bladder (UCC) [75]. In a comparison of miRNA expression levels between clear cell ovarian cancer and normal ovarian surface epithelium, the most downregulated miRNA found was miR-100, which targets FRAP1/mTOR and FGFR3, both of which are effectively pro-growth, pro-cancer proteins [74]. In prostate cancer, miR-100 was also shown to regulate BAZ2, SMARCA5, and THAP2, and was overexpressed in localized *vs.* metastatic disease [68]. Thus, many tumor suppressor proteins and oncogenic products involved in the cell cycle have already been identified as direct or indirect targets of miRNAs, and many more will surely be discovered.

A number of miRNAs are implicated in metastasis but act through yet-unidentified mechanisms. For example, miR-373 may be involved in invasiveness of breast and UCC cancers. This miRNA was first implicated in a large study in which non-metastatic breast cancer cells were transduced with 450 different miRNAs and evaluated each for metastatic properties [80]. Subsequently, miR-373 was found to be significantly upregulated in high-grade, metastatic UCCs as compared to their low-grade counterparts [75].

Epigenetic changes, particularly alterations in the methylation status of DNA coding for miRNA, are likely a leading cause of altered miRNA expression levels in cancer cells. When wild-type colon cancer cells were compared with colon cancer cells subjected to DNA methyltransferase knockout, it was found that the knockout cells contained lower levels of CpG methylation and higher levels of miRNA expression [81]. Since then, aberrant methylation of DNA coding for specific miRNAs has been associated with abnormal levels of those miRNAs in various cancers, including both solid tumors and blood cancers [82]. It is worth noting that not all aberrant miRNA expression appears directly attributable to epigenetics. Diederichs *et al.*, concurrently with Yanaihara *et al.*, found that treating lung cancer cells with demethylating agents and HDACi had no effect on miRNA expression [78,83]. The re-expression of miRNA by epigenetic therapeutics is therefore controversial, but it is possible that the effects are cell line-specific.

Recently, Shen *et al.* described an alternative mechanism of miRNA regulation in tumors [84]. They found that hypoxia, a state common in the center of a solid tumor, enhanced the phosphorylation of argonaute 2 (AGO2) by increasing its association with epidermal growth factor receptor (EGFR). Similarly, bladder cancer cell lines (UCC) subjected to hypoxia exhibited lower levels of miR-100, which targets fibroblast growth factor receptor 3 (FGFR3) [85]. Thus, hypoxia in UCC cells dysregulates miRNA and enhances expression of the pro-cancer FGFR3 protein. These studies demonstrate that changes in the cellular environment can alter miRNA expression levels, ostensibly through non-epigenetic mechanisms.

## 6. Epithelial-Mesenchymal Transition

In epithelial cancers the progression from precursor cells to mature cancer cells is accompanied by an epithelial-mesenchymal transition (EMT). EMT is characterized by a decrease in cell-cell adhesion and an increase in cell motility. The cell-cell attachment receptors are downregulated and the receptors needed for motility are upregulated [86,87]. E-cadherin (E-cad), integrins and their ligands are a few of the examples of such receptors. EMT is also accompanied by the activation/overexpression of surface metalloproteases, which degrade the extracellular matrix, allowing the movement of cells with mesenchymal characteristics, which is necessary for metastasis [86,87].

It has been hypothesized that EMT endows disseminated cancer cells with the ability to overcome systemic dormancy and initiate metastatic outgrowth. This is accomplished by down-regulating E-cad expression or activity, separating cell-cell junctions, invading the surrounding tissues, and intravasating the vasculature or lymphatic system [86,87].

In fully differentiated cells, E-cad functions to maintain cell-cell junctions, thereby inhibiting aberrant cell proliferation and migration. Thus, epigenetic silencing of E-cad is a common characteristic of systemically invasive cancer [88–90]. Recent findings have established E-cad and its response to EMT (induced by TGF-β) as a critical determinant for whether disseminated breast cancer cells acquire dormant or proliferative metastatic programs [91].

Two major cell adhesion molecule families, integrins and selectins, have been identified as participating in metastasis of several types of cancers including colon and lung carcinomas and melanomas [92–95]. Integrins are large, complex, transmembrane glycoproteins which mediate cell adhesion and directly bind components of the extracellular matrix (ECM), such as fibronectin, vitronectin, laminin, or collagen, thereby providing anchorage for cell motility and invasion [96]. Tumor cell expression of the integrins, αvβ3, αvβ5, α5β1, and α6β4, has been correlated with metastatic progression in melanoma, breast carcinoma, prostate, pancreatic, and lung cancer [95].

In addition to the well-established role of integrins during migration and invasion, integrins also regulate other key steps of cancer progression including cancer cell proliferation, survival, and angiogenesis. For example, the ability of breast cancer cells to initiate metastatic outgrowth has recently been linked to the expression and activity of β1 integrin and its downstream effector, focal adhesion kinase (FAK) [91]. These essential mediators of EMT are induced by transforming growth factor-β (TGF-β) in normal and malignant mammary epithelial cells (MECs) [91,97–101].

Ligation of integrins provides survival signals to cancer cells [102]. Downstream signaling of integrins usually works in association with membrane bound or intracellular kinases. The first evidence of β-3 integrin association with Syk-kinase was observed in platelets [103,104]. Interestingly, it is now observed that integrin association with tyrosine kinase receptors is involved in breast cancer progression [100,105]. A recent study also showed that β-3 integrin signaling through Syk-kinase mediates progression of leukemia [106].

Like E-cad, some integrins are silenced by methylation. Examples include α-4-integrin, which is silenced in colon cancer [107], and basement proteins Nidogen 1 and 2 (NID 1 and 2), which regulate integrin function and are silenced in some cancer cells [108]. It has also been found that the expression of αV integrins by neoplastic cells contributes to the promotion of local invasion and metastasis [109]. The most characteristic extracellular ligands of αV integrins are vitronectin and fibronectin. Hepatocytes are the main source of vitronectin. A recent study of hepatocellular carcinoma found that HepG2 and Hep3B cells expressed αV integrin chain and used αVβ1 and αVβ5 for adhesion and migration on vitronectin. Furthermore, tumor necrosis factor (TNF) α and transforming growth factor (TGF) β significantly increased the expression levels of αV integrins and stimulated the adhesion and migration of both HepG2 and Hep3B cell lines on vitronectin [109].

Selectins are vascular cell adhesion molecules involved in adhesive interactions of leukocytes and platelets and endothelium within the blood circulation. There are three members of the selectin family: P-, E-, and L-selectin. Recent evidence indicates that selectin-mediated interactions through cooption of inflammatory pathways contribute to formation of a permissive microenvironment for metastasis [94].

Proteases are often produced by invasive cancer cells as well as by bone marrow-derived cells, including macrophages. These stromal cell-derived proteases include specific cysteine cathepsins [110,111] and serine proteases [112], and matrix metalloproteinases [113,114]. There are several possible mechanisms by which proteases promote cancer cell invasion. They may act as key regulators of cell–cell attachment by cleaving cell-adhesion molecules, such as E-cad, leading to the disruption of cell–cell junctions [111,115]. The loosening of cell contacts facilitates cancer cell migration, either as individual cells or in groups. Protease degradation or turnover of proteins in the ECM and basement membrane enables invasive cells to migrate into the surrounding tissue and vasculature. It is not surprising that elevated levels of distinct proteases, including MMPs, can be detected in tumor tissue or serum of patients with advanced cancer [116]. Alterations within the cytoskeletal architecture also appear necessary to enable dormant breast cancer metastases to reinitiate proliferative programs coupled to metastatic outgrowth [97]. EMT is classically associated with reorganization of the actin cytoskeleton [117].

An EMT can be induced *in vitro* by the transfection and ectopic expression of several transcription factors, such as Twist, Snail, and ZEB1, by treating breast cancer cells with TGF-β, and by the targeted deletion of E-cad in MECs [118–120]. It appears that control of EMT is via signal-transduction pathways such as the Wnt and TGF-β pathways, both of which can be aberrantly activated in neoplastic contexts. One candidate is the *TWIST* gene, described to bind to E-box elements on the *Akt2* promoter and to enhance its transcriptional activity and, thus, is likely to be related to the EMT phenomenon in cancer cells [121–123]. Also involved is PI3Kα, which activates the Akt1 and Akt2 Ser/Thr kinase, responsible for proliferation and antiapoptotic function [124–127].

Epigenetic regulation of EMT also involves miRNAs. For example, possible targets of miR-22 include the ARRB1 protein [74], which is known to activate β-catenin signaling involved in cell–cell adhesion. MiR-22 is downregulated in serous, endometrioid, and clear cell ovarian cancers [128] and reduced expression was associated with gastric cancer metastases [129]. However, miR-22 has also recently been shown to promote metastasis in a transgenic mouse breast cancer model by silencing TET-mediated demethylation of anti-metastatic miR-200 [130,131]. Clearly, the functions of miR-22 are complex and likely context-dependent, but the involvement of miR-22 in EMT and metastasis is certain.

MiR-126 and miR-335 have also been identified as anti-metastatic miRNAs that are significantly downregulated in breast cancer patients [132]. When these miRNAs were re-expressed in cancer cells *in vivo*, the incidence of lung and bone metastases decreased. It has been suggested that CBX7 positively regulates E-cadherin [133]. A knockdown of miR-182 *in vitro* led to upregulation of CBX7 and E-cadherin [77] in breast cancer cells. These results suggest that the overexpression of miR-182 is at least partially responsible for invasiveness of certain cancers through its role in facilitating the EMT.

In addition to accumulating the changes associated with the EMT, an invasive cell must break through a basement membrane in order to metastasize to new locations in the body. Therefore, a compromised basement membrane near a primary tumor increases the likelihood of metastasis. Interestingly, miR-205 has been showed to be involved in a regulatory network responsible for the deposition of the basement membrane in prostatic epithelium [134]. Loss of this miRNA may compromise the basement membrane and facilitate metastasis of prostate cancer.

## 7. A Model for Epigenetics in Carcinogenesis, Progression, and Metastasis

Recent studies suggest that cancer progression occurs from cancer stem cells. Weinberg *et al.* postulated that a few of the cancer stem cells in a population of cancer cells forming a benign tumor acquire metastatic potential by intrinsic or induced mechanisms [135]. Induced mechanisms usually occur by reactive stroma. The metastatic cancer stem cells (CTCs) then transit to distant organs. We hypothesize that a mixture of metastatic cancer cells and metastatic cancer progenitor cells travel to different organs (Figure 1A, f). We also discuss the possible way these progenitor cells are formed. Theoretically, the progression of cancer and acquisition of metastatic potential requires differentiation of these cancer stem cells. We propose that epigenetic and other changes mediate the development of cancer progenitor cells from cancer-predisposed cells (Figure 1A, a and b) [5], and that epigenetic mechanisms are also critical for epithelial-mesenchymal transition (EMT).

The concept of “cancer stem cells” has existed for more than a decade, but how they develop remains a mystery. For clarity, we prefer the term “cancer progenitor cells” rather than “cancer stem cells”. The stem-like properties of a cancer progenitor cell are more analogous to that of an induced pluripotent stem cell (iPSC) than to that of an embryonic stem cell (ESC). Cancer progenitor cells are the earliest form of cancer cells. These cells have acquired insensitivity to growth regulators via silencing of apoptotic or autophagic mechanisms, and may initiate local tumorigenesis. However, metastasis requires differentiation of a subpopulation of cancer progenitor cells into a metastatic form before outgrowth of metastases can occur (Figure 1A, d and e). The best example of this type of differentiation is the EMT. Most EMT studies concentrate on endpoints, in which cells exhibit either epithelial or mesenchymal characteristics, as described in the previous section. However, the process by which this transition occurs is not as well defined. *In vitro* studies show that TGF-β and three families of transcription factors, ZEB, Snail, and Twist, play a significant role in the EMT [136]. A large number of signaling molecules, other transcription factors, and miRNAs also play a role in this transition [137].

We hypothesize that the transformation of cancer progenitor cells to metastatic cancer progenitor cells occurs before rapid cell growth (Figure 1A, e). Conceptually, differentiation and cell growth are antagonistic. Epithelial cancer progenitor cells must undergo a transformation to mesenchymal cells, triggered by signaling mechanisms that may involve TGF-β and various transcription factors. During this transition, cells must survive and divide but are not rapidly growing. In addition to promoting differentiation, TGF-β is also known to induce apoptosis. However, during cancer progenitor cell differentiation, the downstream effectors that mediate the pro-apoptotic role of TGF-β are inhibited. A recent study shows that TGF-β-induced EMT allows cell cycle progression but inhibits apoptosis [138]. The induction of differentiation, as well as the survival mechanism, may involve intracellular, epigenetic, and stromal cell signals (Figure 1A, c and d). The survival signal could be a downstream effect of integrin ligation [102]. Once differentiation progresses to the point at which the EMT is almost complete, the transformed cancer progenitor cells trigger the activation and overexpression of proliferative genes and deactivate differentiation genes (Figure 1A, d and e). This stepwise progression is corroborated by discrete, grade-specific cancer cells found in patients.

The development of grade-specific cancers can be explained by this model (Figure 1B). The differentiation of epithelial cancer progenitor cells to the mesenchymal form of progenitor cells is a multi-step process, and cancer progenitor cells are not synchronized in development. One possibility is that some cells will progress further through differentiation than others, stop differentiation, and then proliferate, giving rise to clonal populations of cancer cells at distinct grades (Figure 1C). The more plausible explanation is that cancer progenitor cells may pause at each grade of differentiation, and proliferate from that grade while maintaining the ability to differentiate further (Figure 1B).

For a cancer progenitor cell to pause at a particular grade and proliferate, genes for proliferation must be activated and genes for differentiation must be inactivated. Epigenetic regulation is well positioned to mediate this switching mechanism. This hypothesis is supported by the recent discovery that epigenetic suppression of TGF-β signaling was observed in metastatic ovarian cancers [139]. In another study, methylation of the genes for TGF-β receptors 1 and 2 is more frequent in grade III/IV than in grade I/II esophageal squamous cell carcinoma, indicating that epigenetic regulation of this pathway is critical for cancer progression [140].

Another important consideration is that overgrown metastatic cancer cells and metastatic cancer progenitor cells need to colonize at the distant site before they may overgrow. This issue is also discussed by Chaffer and Weinberg [135]. They have suggested that metastasized cells at distant organs must adapt to permit localization. We believe that those cells go through a partial mesenchymal-epithelial transition (MET), allowing expression of cell–cell adhesion receptors. This process likely involves the reversal of distinct epigenetic changes in response to stromal cell signaling. The characteristics necessary for partial-MET are perhaps context- or cancer- dependent, as it has been observed that lung cancer metastasis is faster than metastasis of breast and prostate cancers.

## 8. Clinical Aspects of Cancer and Therapeutics

The development of cancer therapeutics is challenging given inter-patient, and even intra-patient, heterogeneity. Therapies targeting well-defined markers, such as overexpressed Her-2 in breast cancer or fused Bcr-abl in CML, are often initially successful but falter when subpopulations of resistant cancer cells become dominant. The new paradigm of drug development involves targeting multiple hallmarks of cancer simultaneously. We have proposed that exposure to epigenetic and non-epigenetic drugs which re-express tumor suppressor genes should sensitize the cancer cells to lower doses of traditional cytotoxic drugs [5]. Recent studies support this hypothesis. For example, treatment with HDACi sensitizes breast and ovarian cancer cell lines to the calpeptin, TRAIL, and telomere homolog oligonucleotides [60,141,142]. The demethylating agent, 5-azacitidine, sensitizes ovarian cancer cells to classical platinum-based chemotherapeutics [143]. In most of these examples, the combination drug treatment induces cell death selectively in cancer cells, through mechanisms that likely involve apoptosis and autophagy. A recent study showed that telomere homolog oligonucleotides re-express the death receptors DR-4 and DR-5 in ovarian cancer cells. Combination treatment with TRAIL induced apoptosis in the oligonucleotide-resistant ovarian cancer cells [60].

The prerequisite of the re-expression of epigenetically silenced tumor suppressor genes is demethylation of the regulatory regions. Though DNA methyltransferase-1 (DNMT1) inhibitors, such as 5-azacitidine and its derivatives, are the most well-known demethylating agents, recent studies have also shown that HDACi demethylates regulatory regions of silenced tumor suppressor genes in cancer cells via downregulation of DNMT1 [5,17,144,145]. A possible role of an as yet unidentified demethylase is postulated in the drug induced rapid demethylation process [5]. Demethylation of the regulatory regions of tumor suppressors such as p16, p21, RAR-β2, or ARHI results in variable re-expression, with levels dependent on inhibitor type and cell line [5,17,144]. Though the effects of HDACi are variable, HDACi combination with 5-azacitidine elicits synergistic-type demethylation compared to individual treatment [146]. The recent observation that HDACi, which were originally intended to increase histone acetylation levels, are also able to induce demethylation of CpGs increases the potential of HDACi as epigenetic therapeutics.

Many of the oncogenes implicated in carcinogenesis are kinases that become constitutively activated or overexpressed, leading to increased phosphorylation levels of important regulatory proteins. For example, overexpression of HER-2 in breast cancer activates ERK, Akt, PLCγ, PKC, and STAT signaling pathways, which leads to proliferation, inhibition of apoptosis, and adverse outcomes in clinical scenarios [147]. Recent results suggest that DNMT1 is regulated by ERK kinase, indicating a pathway by which aberrant signaling may give rise to epigenetic modifications in carcinogenesis [144]. Other studies have shown that Akt-dependent phosphorylation may also regulate DNMT1 activity [148,149]. Pradhan *et al.* [148,149] showed that DNMT1 is stabilized by phosphorylation by Akt. Zuo *et al.* have shown that inhibition of Akt demethylates key silenced genes [148]. The demethylation process is likely by the down-regulation of DNMT1. Further research is needed to fully understand how upstream signaling regulates DNMT1 in the context of carcinogenesis. However, these results may begin to guide the development of inhibitors specific to methylation signaling molecules. In addition to specific DNMT1 inhibitors such as AZA and its derivatives, signaling inhibitors could hold promise as future epigenetic therapeutics for cancer.

MicroRNA is another important epigenetic regulatory system that may be targeted as cancer therapy. Targeting specific miRNAs could be particularly effective in cancers with miRNAs found to confer chemotherapeutic resistance. For example, though paclitaxel is the standard chemotherapeutic administered for advanced cervical cancer, resistance against this drug remains high and survival rates low. It has been observed paclitaxel upregulates miR-375 in a dose-dependent manner, and that overexpression of miR-375 increases resistance to paclitaxel *in vitro* and *in vivo* [150]. Thus, miR-375 interference or destruction is a promising therapeutic avenue in the context of paclitaxel-resistant cervical cancer. Similarly, miR-30c, miR-130a, and miR-335 have been shown to be consistently downregulated in drug-resistant ovarian cancer, and it has been demonstrated that the well-described resistance factor M-CSF is downstream of miR-130a [151]. These miRNAs and their downstream targets and associated pathways may represent excellent targets in the fight against drug-resistant cancers.

Already some epigenetic therapies have been shown to be effective in fighting cancer in clinical settings. For example, DNA methyltransferase inhibitors 5-azacytidine (azacytidine) and its deoxyribose analog, 5-aza-2′-deoxycytidine (decitabine), are both FDA approved for treatment of myelodysplastic syndromes. Treatment of solid tumors with the maximum dose of these compounds led to extensive toxicity and minimal efficacy, but lower concentrations effectively reversed tumor-specific DNA methylation [152].

In a study of non-small cell lung cancers (NSCLC), an association was observed between the methylation status of four genes, *p16*, *CDH13*, *APC*, and *RASSF1A*, and the probability of post-treatment recurrence [153]. Methylation of the promoter region of these genes was present even in histologically normal lymph nodes of recurrent patients, a finding that was attributed to otherwise undetectable micrometastases. We believe that the main constituents of these micrometastases are cancer progenitor cells in the pre-proliferative stages of metastasis. These cells would require further differentiation and passage through MET to become a metastatic cancer capable of rapid growth, as described in Figure 1, which would be clinically observed as recurrence. This perspective further encourages the use of epigenetic therapies in the context of resistant or recurrent cancer. Epigenetic therapies may help to target disseminated cancer progenitor cells by reversing some of the epigenetic changes that make this population of cells so resistant to traditional chemotherapeutics.

A recent phase I/II clinical trial of a combination therapy of azacitidine and entinostat (class 1 HDAC inhibitor) in patients with recurrent metastatic NSCLC has shown that combination epigenetic therapy has efficacy and is well tolerated [154]. The median progression-free survival was 7.4 weeks, and the median overall survival among patients who completed at least one epigenetic therapy cycle was 8.6 months. Promoter methylation status was determined for the genes previously found hypermethylated in recurring NSCLC (APC, RASSF1A, CDH13, CDKN2A) at pre- and post-treatment [153,154]. Ten patients had at least two methylated genes (methylation-positive) pre-treatment and showed a decrease in methylation levels of two or more of these genes post-treatment. Eight of these ten patients had either stable disease or objective responses to epigenetic therapy. The remaining patients in the study were methylation-negative at the identified loci and had no objective responses to treatment. Finally, four patients that received immediate subsequent chemotherapy had major objective responses, supporting the proposed synergistic effects of a combination epigenetic and chemotherapeutic treatment plan.

Another phase I study examined HDACi in combination with chemotherapy in patients with relapsed or refractory leukemia. It was anticipated that Vorinostat, an HDACi approved for persistent cutaneous T cell lymphoma, could sensitize cancer cells to idarubicin, in accordance with the synergistic effect observed in a preclinical study [155]. Overall, 17% of patients had a response to this combination treatment, and two patients had a complete response. Histone acetylation measurements taken from 33 of 41 patients revealed that 46% had increased acetylation. Upregulation of the HDACi-associated kinase inhibitor, CDKN1A, was observed; however, it was not clear if this effect was due to Vorinostat or idarubicin, which is also known to induce CDKN1A.

Two other phase I studies of leukemia studied the effects of decitabine alone [156] and in combination with valproic acid [157]. Dose-limiting myelosuppression prevented dose escalation of decitabine to levels associated with global methylation changes in the treatment of chronic lymphocytic leukemia and non-Hodgkin lymphoma [156]. However, in the context of acute myeloid leukemia, low-dose decitabine was found to be safe for eliciting promoter demethylation, depletion of DNMT1, and histone hyperacetylation, leading to a clinical response rate of 52% [157]. Four patients demonstrated complete remission and another seven patients demonstrated incomplete or partial remission. The addition of valproic acid, however, led to the development of encephalopathy at relatively low doses.

These clinical studies suggest that combination treatment with epigenetic drugs and standard chemotherapy is a powerful treatment paradigm that is capable of potentiating classical treatments and reducing relapse in the context of many different types of cancer. It is possible that these types of therapy are more effective because they kill progenitor cancer cells. Further studies will reveal the exact mechanisms of how these epigenetics therapies elicit better outcomes.

## 9. Conclusions

This review summarizes the available literature on the role of epigenetic alterations as observed in many different cancers. We have also provided a perspective on the generation of metastatic progenitor cancer cells from precursor cancer progenitor cells. Many epigenetic changes, such as hypomethylation of oncogenes, hypermethylation of tumor suppressor genes, depletion of hydroxymethylation, changes of histone acetylation and methylation patterns, and miRNA expression level variations, are known to be associated with many cancers. Further studies are expected to elucidate how these variations are generated and, in turn, how they mediate the development of metastatic cancer progenitor cells. The knowledge of this mechanism is not only important to understand how cancer cells transform and acquire resistance to chemotherapy, but will be invaluable in the design of more potent epigenetic drugs. These treatments, in combination with traditional therapies such as surgery, radiation, and traditional chemotherapy, will permit targeting of cancer progenitor cells and likely reduce the significant mortality associated with cancer relapse.
